# Comparison of Pentax-AWS Airwayscope and Glidescope for Infant Tracheal Intubation by Anesthesiologists during Cardiopulmonary Arrest Simulation: A Randomized Crossover Trial

**DOI:** 10.1155/2015/190163

**Published:** 2015-06-16

**Authors:** Shunsuke Fujiwara, Nobuyasu Komasawa, Sayuri Matsunami, Daisuke Okada, Toshiaki Minami

**Affiliations:** Department of Anesthesiology, Osaka Medical College, Osaka 569-8686, Japan

## Abstract

*Background*. Recent guidelines for infant cardiopulmonary resuscitation emphasize that all rescuers should minimize interruption of chest compressions, even for endotracheal intubation. We compared the utility of the Pentax-AWS Airwayscope (AWS) with the Glidescope (GS) during chest compressions on an infant manikin. *Methods*. Twenty-four anesthesiologists with more than two years of experience performed tracheal intubation on an infant manikin using the AWS and GS, with or without chest compressions. *Results*. In GS trials, none of the participants failed without compressions, while three failed with compressions. In AWS trials, all participants succeeded regardless of chest compressions. Intubation time was significantly longer with chest compressions with the GS (*P* < 0.05), but not with the AWS. Difficulty of operation on a visual analog scale (VAS) for laryngoscopy did not increase significantly with chest compressions with either the GS or the AWS, while the VAS for tube passage through the glottis increased with compressions with the GS, but not with the AWS. *Conclusion*. We conclude that in infant simulations managed by anesthesiologists, the AWS performed better than the GS for endotracheal intubation with chest compressions.

## 1. Background

The European Resuscitation Council (ERC) cardiopulmonary resuscitation (CPR) guidelines emphasize the paramount importance of minimizing chest compression interruptions to maximize cerebral and coronary perfusion pressure [1]. Moreover, the guidelines recommend that skilled rescuers should secure the airway without interrupting chest compressions or with only a brief pause to visualize vocal cords and allow passage of the tracheal tube [2].

Direct laryngoscopy with the Miller laryngoscope (Mil) is the most widely used technique for infant tracheal intubation. However, the Mil can be difficult to use even for skilled professionals and could become detrimental in infant emergent situations [3]. Previous studies reported that the Pentax-AWS Airwayscope (AWS; Hoya, Tokyo, Japan) was a more functional device alternative than the conventional Mil for intubation during chest compressions [4, 5].

The Glidescope Cobalt (GS; Verathon Medical, Washington, USA) is a video laryngoscope reported to provide a nonsightline view of the airway. Various clinical and simulation studies indicate that the GS is not only useful for difficult airway management in adults [6–8]. Furthermore, utility of GS for emergent tracheal intubation with chest compressions in adult simulations or clinical study has been suggested [9–11]. Both the AWS and GS provide a nonsightline view and are considered convenient tools for emergent tracheal intubation in adults.

An infant-sized Intlock blade was recently developed for the GS and the utility for emergency airway management by pediatric fellows or novice doctors has been evaluated [12–14]. The result showed GS was inferior to Mil for emergency tracheal intubation in infants or neonates. All reports suggest that the GS inferiority was partially attributed to the small clinical airway management experience with novice doctors or pediatricians [8, 12–14]. Based on these previous reports, we considered that definite evaluation of GS during infant chest compression by anesthesiologists, who specialize and routinely perform airway management, is needed.

Comparison of GS and AWS utility by anesthesiologists during infant chest compression has not been validated. Therefore, we decided to compare the utility of AWS to the infant-size GS for anesthesiologists. We hypothesized that the AWS or GS would improve intubation in simulations with chest compressions. In the present study, we compared AWS and GS performance with respect to ease of tracheal intubation by anesthesiologists during chest compressions on an infant manikin.

## 2. Methods

From May to August 2014, 24 anesthesiologists who had more than two years of experience were recruited from our institute or medical personnel taking an anesthesiology training course at the Osaka Medical College. Selected participants had 4.8 ± 2.8 years of clinical experience in anesthesia. Written informed consent was obtained before the study and participants were asked for previous clinical experience with AWS or GS. This study was approved by the Osaka Medical College Research Ethics Committee.

**Figure 1:**
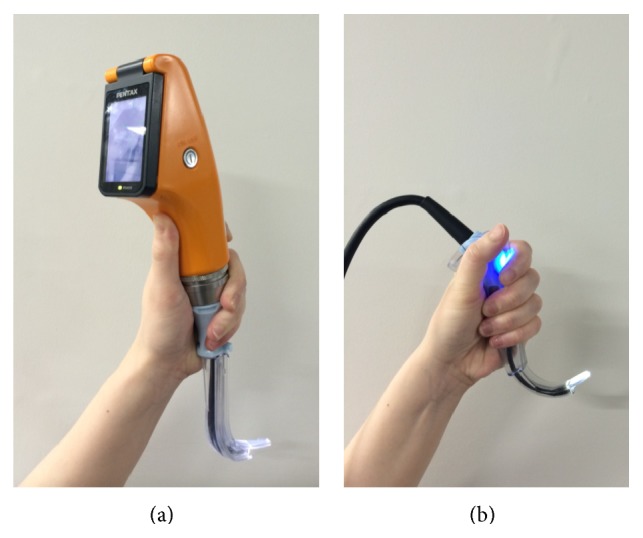
The two video laryngoscopes for infants used in the study. (a) Pentax-AWS Airwayscope with an infant-sized Intlock blade; (b) Glidescope with size 1 blade.

The ALS Baby Trainer manikin (Laerdal, Stavanger, Norway), designed to accurately represent a three-month-old infant (weight: 11 pounds), was used in the study simulations to perform intubations and chest compressions [15]. Participants used a tracheal tube (Portex, St. Paul, MN, USA) without a cuff and with an internal diameter of 3.5 mm, as well as the AWS and the infant GS with a size 1 blade (Figure 1).

The manikin was placed on a hard, flat table for “on the bed” simulation. Chest compressions were performed by the same Basic Life Support instructor using the two-thumb technique at a depth of about two inches and a rate of 100 compressions per minute in accordance with present guidelines.

Each participant was instructed to insert the tracheal tube, attach a bag valve mask, and attempt to ventilate the lungs of the manikin. Participants were given ten minutes to practice intubation, with the instructor available to give advice. The appropriate equipment for each trial was placed in a box next to the manikin's head. Intubation started when the participant picked up the AWS or GS and ended at the point of manual ventilation after tube insertion. Intubation times were recorded for both tracheal and esophageal intubations. For chest compression trials, participants were not allowed to discontinue compressions. At the end of the study, participants rated the difficulty of using each device for laryngoscope imaging and passage of the tracheal tube through the glottis on a visual analog scale (VAS) from 0 mm (extremely easy) to 100 mm (extremely difficult) [16].

Results obtained from each trial were compared using two-way repeated measures analysis of variance for intubation time and VAS and Fisher's exact test for the success rate. Clinical experience of AWS and GS was compared with Mann-Whitney *U* test. Data are presented as mean ± SD. *P* < 0.05 was considered statistically significant.

The study was designed as a randomized crossover trial to minimize the learning-curve effect. The order of intervention was randomized for each participant using the random number table, resulting in a total of four interventions per participant (24 patterns).

Results of a nine-doctor preliminary study showed that the time required to ventilate lungs after successful insertion of the AWS was approximately 14 ± 4 s. We estimated that 22 participants would be adequate for two independent groups using *α* = 0.05 and *β* = 0.2.

## 3. Results

Clinical experience of number of the participants with the AWS was significantly higher than that with GS (AWS 60.2 ± 40.8 times versus GS 30.2 ± 20.2 times, *P* < 0.05). All participants had the experience of these two devices more than 10 cases.

### 3.1. Endotracheal Intubation Success with GS or AWS

The number of successful tracheal intubations for each device is displayed in Table 1. With the GS, no participant failed to achieve intubation without chest compressions, and three failed with compressions (N.S.). With the AWS, all intubations were successful regardless of whether chest compressions were performed.

### 3.2. Intubation Time with GS or AWS

**Figure 2:**
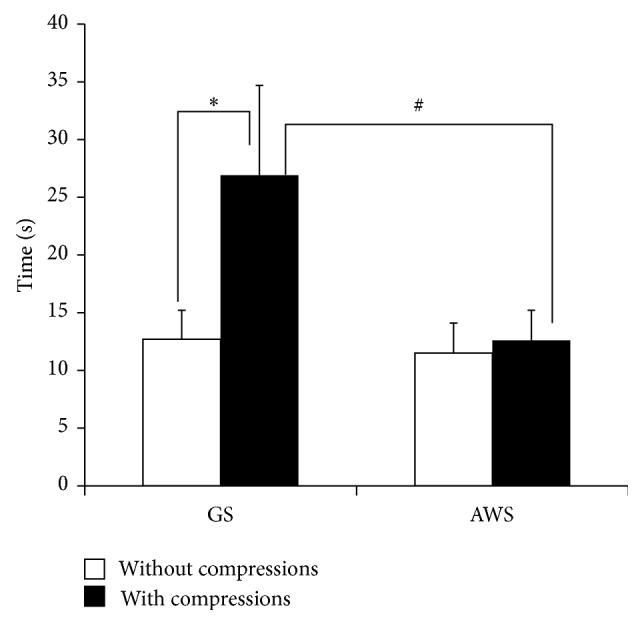
Time elapsed for simulated infant tracheal intubation with and without chest compressions between GS and AWS. GS: Glidescope; AWS: Pentax Airwayscope with an infant-sized Intlock. Results are expressed as mean ± SD and analyzed with two-way analysis of variance. ^*∗*^ *P* < 0.05 compared to chest compressions. ^#^ *P* < 0.05 compared to AWS.

With the GS, tracheal intubation took significantly longer with chest compressions (26.9 ± 7.8 s) than without compressions (12.7 ± 2.5 s; *P* < 0.05) (Figure 2). In contrast, chest compressions increased intubation time slightly, but not significantly, with the AWS (with compressions, 12.6 ± 2.6 s; without compressions, 11.5 ± 2.6 s).

Intubation time without chest compressions was not significantly longer with the GS than AWS. However, the intubation time was significantly shorter with chest compressions with the AWS than with the GS (*P* < 0.05).

### 3.3. VAS Scores for Laryngoscopy and Tube Passage through the Glottis for GS or AWS

**Figure 3:**
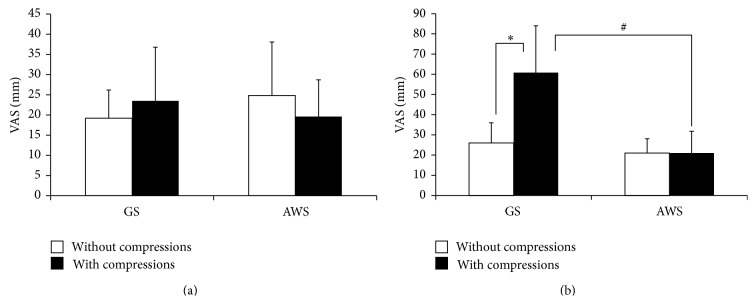
Visual analog scale for simulated infant tracheal intubation with and without chest compressions between GS and AWS. (a) Laryngoscope image; (b) passage of the tube through the glottis. GS: Glidescope; AWS: Pentax Airwayscope with an infant-sized Intlock. Results are expressed as mean ± SD and analyzed with two-way analysis of variance. ^*∗*^ *P* < 0.05 compared to chest compressions. ^#^ *P* < 0.05 compared to AWS.

As shown in Figure 3, although the VAS score for laryngoscopy was not significantly higher with the GS with chest compressions, the score for tube passage through the glottis was significantly worsened by chest compression. With the AWS, neither VAS score was significantly affected by chest compressions.

VAS scores for laryngoscopy were not significantly different between the AWS and GS. Scores for tube passage through the glottis were significantly lower with chest compressions with the AWS than with the GS (*P* < 0.05).

## 4. Discussion

Current ERC guidelines emphasize the administration of continuous chest compressions with as few interruptions as possible, including short pauses for airway management [1, 2]. As asphyxia is the most common cause for cardiac arrest in infants, not only continuous chest compression but also rapid and successful airway management is the most important during infant resuscitation [17, 18]. From this viewpoint, airway management such as tracheal intubation is critical for infant CPR. Although the most widely used laryngoscope for these situations is the direct Miller laryngoscope, its difficulty to operate without experience can lead to an unacceptably high incidence of inaccurate intubation [3].

The GS offers accurate visualization of the glottis with clear laryngeal exposure compared to the conventional direct laryngoscope, as it utilizes indirect laryngoscopy and higher magnification. Prior studies have demonstrated that the GS reduces the difficulty of tracheal intubation in direct comparisons with the conventional Macintosh laryngoscope [7–9].

The AWS is a video laryngoscope designed to provide a clear view of the glottis and its surrounding structures. The nonsightline view characteristics of the AWS improve the laryngeal view compared to other laryngoscopes, and its tube guide facilitates rapid and reliable tracheal intubation even for difficult adult cases involving issues such as cervical neck immobility or morbid obesity [19, 20]. Evidence indicates that the AWS is also suitable for difficult airway management and emergent situations and that it is easy for novice doctors to use [21]. Previous studies reported that the AWS with an infant-sized Intlock requires less skill and is well suited for those who perform infrequent intubations in emergency situations [4].

In the present study, we demonstrated that the success rate of intubation with the GS decreased during chest compressions, with a significant increase in intubation time. Intubation time did not significantly increase with the AWS, and all anesthesiologists achieved successful intubation during chest compressions. One probable reason for difficulties experienced with the GS is that the glottis, but not the tube, moved during chest compressions, and the relative positions of the glottis and tube were thus unstable. We speculate that this is the underlying reason for difficulties with the GS. With a nonsightline laryngoscope with a tube guide like the AWS, however, the tube and glottis could move simultaneously while their relative positions remained the same, leading to easy and safe tracheal intubation.

VAS scores for both laryngoscopy and tube passage through the glottis with chest compressions differed significantly between the GS and AWS, with the AWS providing easier laryngoscopy and tube passage. Features of the AWS, such as the target mark, may have contributed to the quick and accurate tube passage through the glottis. Another probable reason for the success of the AWS is the presence of a target mark and built-in conduits. Once the target mark on the AWS is aligned with the glottis as shown in the monitor, the tracheal tube can be pushed through the vocal cords. Although the GS is an effective infant intubation tool, the AWS appears to be more suitable for airway management by anesthesiologists performing chest compressions.

Difficult airway management for infants includes specific physical difficulties, such as a small jaw and subglottic narrowing [22]. Intubation is also complicated by the different location and administration of chest compressions compared to an adult [23, 24]. The AWS would prove useful for prompt and reliable infant airway rescue even in emergent situations.

This study has several limitations worth noting. First, the simulations do not account for factors such as blood, vomit, or secretions in the oropharynx; they also do not include the risk of blurred images due to fogging of the AWS or GS monitor. Second, the AWS and GS were not evaluated in difficult infant airways and the results cannot be extrapolated to other patient situations involving issues such as severely restricted mouth opening. Third, results of VL by experienced operators such as anesthesiologists cannot be extrapolated to other pediatric care settings because most CPR are performed by prehospital care providers and pediatricians. Trials for AWS and GS utility evaluation by prehospital care provider or pediatrician are needed in the future. Fourth, the clinical experience of participants with AWS was significantly higher than that with GS, which may have affected the results. Fifth, chest compressions and intubation were performed on an infant manikin, which leads to shorter airway intervention times than that required for actual patients [25]. Finally, homogeneity of CPR techniques cannot always be assured in clinical situations.

Clinical experience accumulation and randomized trials of AWS and GS use with actual patients receiving CPR are needed in the future.

## 5. Conclusion

We conclude that the AWS performed better than the GS for endotracheal intubation with chest compressions in infant simulations managed by anesthesiologists.

## Figures and Tables

**Table 1:** Tracheal intubation success rates for GS or AWS with and without chest compressions. GS: Glidescope; AWS: Pentax Airwayscope with an infant-sized Intlock.

	Without chest compressions (successful/total)	With chest compressions (successful/total)	*P* value (Fisher's exact test)
AWS	24/24	24/24	1.00
GS	24/24	21/24	0.23
*P* value (Fisher's exact test)	1.00	0.23	

Numerator: number of participants who successfully intubated. Denominator: number of participants who attempted tracheal intubation. Differences were analyzed with Fisher's exact test. ^*^ *P* < 0.05.

## Abbreviations

CPR: Cardiopulmonary resuscitation
ERC: European Resuscitation Council
AWS: Pentax-AWS Airwayscope
GS: The Glidescope Cobalt.
